# Raw Eggs To Support Postexercise Recovery in Healthy Young Men: Did Rocky Get It Right or Wrong?

**DOI:** 10.1093/jn/nxac174

**Published:** 2022-08-09

**Authors:** Cas J Fuchs, Wesley J H Hermans, Joey S J Smeets, Joan M Senden, Janneau van Kranenburg, Stefan H M Gorissen, Nicholas A Burd, Lex B Verdijk, Luc J C van Loon

**Affiliations:** Human Biology, School of Nutrition and Translational Research in Metabolism (NUTRIM), Maastricht University Medical Centre+, Maastricht, The Netherlands; Human Biology, School of Nutrition and Translational Research in Metabolism (NUTRIM), Maastricht University Medical Centre+, Maastricht, The Netherlands; Human Biology, School of Nutrition and Translational Research in Metabolism (NUTRIM), Maastricht University Medical Centre+, Maastricht, The Netherlands; Human Biology, School of Nutrition and Translational Research in Metabolism (NUTRIM), Maastricht University Medical Centre+, Maastricht, The Netherlands; Human Biology, School of Nutrition and Translational Research in Metabolism (NUTRIM), Maastricht University Medical Centre+, Maastricht, The Netherlands; Human Biology, School of Nutrition and Translational Research in Metabolism (NUTRIM), Maastricht University Medical Centre+, Maastricht, The Netherlands; Human Biology, School of Nutrition and Translational Research in Metabolism (NUTRIM), Maastricht University Medical Centre+, Maastricht, The Netherlands; Human Biology, School of Nutrition and Translational Research in Metabolism (NUTRIM), Maastricht University Medical Centre+, Maastricht, The Netherlands; Human Biology, School of Nutrition and Translational Research in Metabolism (NUTRIM), Maastricht University Medical Centre+, Maastricht, The Netherlands

**Keywords:** muscle protein synthesis, resistance exercise, anabolism, egg protein, cooked eggs, boiled eggs, food processing, digestion, absorption, preheating

## Abstract

**Background:**

Egg protein is ingested during recovery from exercise to facilitate the postexercise increase in muscle protein synthesis rates and, as such, to support the skeletal muscle adaptive response to exercise training. The impact of cooking egg protein on postexercise muscle protein synthesis is unknown.

**Objectives:**

We sought to compare the impact of ingesting unboiled (raw) compared with boiled eggs during postexercise recovery on postprandial myofibrillar protein synthesis rates.

**Methods:**

In a parallel design, 45 healthy, resistance-trained young men (age: 24 y; 95% CI: 23, 25 y) were randomly assigned to ingest 5 raw eggs (∼30 g protein), 5 boiled eggs (∼30 g protein), or a control breakfast (∼5 g protein) during recovery from a single session of whole-body resistance-type exercise. Primed continuous l-[*ring*-^13^C_6_]-phenylalanine infusions were applied, with frequent blood sampling. Muscle biopsies were collected immediately after cessation of resistance exercise and at 2 and 5 h into the postexercise recovery period. Primary (myofibrillar protein synthesis rates) and secondary (plasma amino acid concentrations) outcomes were analyzed using repeated-measures (time × group) ANOVA.

**Results:**

Ingestion of eggs significantly increased plasma essential amino acid (EAA) concentrations, with 20% higher peak concentrations following ingestion of boiled compared with raw eggs (time × group: *P* < 0.001). Myofibrillar protein synthesis rates were significantly increased during the postexercise period when compared with basal, postabsorptive values in all groups (2–4-fold increase: *P* < 0.001). Postprandial myofibrillar protein synthesis rates were 20% higher after ingesting raw eggs [0.067%/h; 95% CI: 0.056, 0.077%/h; effect size (Cohen *d*): 0.63], and 18% higher after ingesting boiled eggs (0.065%/h; 95% CI: 0.058, 0.073%/h; effect size: 0.69) when compared with the control breakfast (0.056%/h; 95% CI: 0.048, 0.063%/h), with no significant differences between groups (time × group: *P* = 0.077).

**Conclusions:**

The ingestion of raw, as opposed to boiled, eggs attenuates the postprandial rise in circulating EAA concentrations. However, postexercise muscle protein synthesis rates do not differ after ingestion of 5 raw compared with 5 boiled eggs in healthy young men.

This trial was registered at the Nederlands Trial Register as NL6506 (www.trialregister.nl).

## Introduction

“… Rocky's alarm clock goes off at exactly 4 a.m. Not accustomed to rising this early, with great difficulty Rocky staggers to his feet and wavers to the bathroom. He turns the light on and roaches scatter. At the top of the mirror hang the telegrams. Rocky fills the basin and submerges his face in cold water. Rocky sways to the icebox and removes a dozen eggs. He cracks five raw eggs into a glass and downs it in one swill… his body quivers. Rocky steps outside. He is dressed in a well-worn sweat suit with a hood, gloves and sneakers. It is pitch dark and his steaming breath attests to the cold. He begins running down the center of the deserted street…” (1). This iconic scene of the 1976 movie Rocky has been imprinted in the brain of many young and older athletes and has been an early sign of the recognition of dietary protein consumption as an important factor in supporting the anabolic response to exercise. However, how much evidence is there for the use of ingesting raw eggs to optimize recovery from training? Scientific evidence has previously shown that raw eggs do not show the same digestion and absorption kinetics when compared with cooked eggs (2, 3). In fact, raw eggs have been reported to show merely ∼51% protein digestion and amino acid (AA) absorption, whereas cooked eggs show ∼91% protein digestion and AA absorption (2). This clearly demonstrates that food processing (such as heat treatment) strongly influences protein digestibility. Indeed, previous work has shown that heat treatment is an important factor determining protein digestion, AA absorption, and subsequent postprandial plasma AA availability (4, 5).

It is well known that protein digestion and AA absorption and the splanchnic extraction of dietary protein-derived AAs are of key importance in driving the postprandial skeletal muscle adaptive response. Specifically, rapid protein digestion rates facilitate a strong increase in postprandial plasma AA availability to stimulate muscle protein synthesis (6–10). Hence, protein ingestion during recovery from exercise helps athletes to further augment muscle protein synthesis rates and, as such, is frequently applied to facilitate the conditioning process following exercise training.

The purpose of this study was to investigate the effectiveness of eating raw eggs, as depicted in Rocky's iconic cinematic scene, to support skeletal muscle conditioning. We hypothesized that the postprandial rise in circulating AAs would be attenuated after the ingestion of raw eggs when compared with boiled eggs, thereby lowering the postprandial stimulation of muscle protein synthesis. If true, this would suggest that Rocky could have done better by boiling his eggs. However, to date, no study has compared the impact of ingesting raw, unboiled eggs with ingesting boiled eggs on postprandial plasma AA concentrations and muscle protein synthesis rates during recovery from exercise. In the present study, we recruited 45 healthy young men to assess the postprandial increase in plasma AA concentrations and the subsequent increase in skeletal muscle protein synthesis rates following ingestion of 5 raw eggs, 5 boiled eggs, or a control breakfast during recovery from exercise.

## Methods

### Subjects

Forty-five healthy, resistance-trained young men (age 24 y; 95% CI: 23, 25 y) participated in this randomized controlled intervention study. This study was conducted between November 2017 and March 2019 at Maastricht University Medical Centre+, in Maastricht, the Netherlands (for CONSORT flow chart, please see **Supplemental Figure 1**). Subjects’ characteristics are presented in **Table 1**. Subjects were fully informed of the nature and possible risks of the experimental procedures before their written informed consent was obtained. Participants were eligible to participate if they were males aged 18–35 y, had a BMI of 18.5–30 kg/m^2^, were nonsmokers, were deemed to be healthy (i.e., free of cardiovascular, musculoskeletal, or metabolic conditions), were not taking any medication, did not have any food allergies to eggs, and were familiar with performing resistance-type exercise training (≥2 times per week). This study was approved by the Medical Ethics Committee of Maastricht University Medical Centre+ (METC 173030) and conforms to the principles outlined in the Declaration of Helsinki (of 1975 as revised in October 2013) for use of human subjects and tissue. This trial was registered at https://english.ccmo.nl/latest/news/2022/06/24/dutch-trial-register-ntr-no-longer-available(no. NL6506).

**TABLE 1:** Subjects’ characteristics^1^

	Control breakfast	Raw eggs	Boiled eggs	*P*
Age, y	23 (22, 25)	24 (22, 26)	24 (22, 26)	0.587
Body mass, kg	75.2 (71.1, 79.3)	79.8 (75.6, 83.9)	76.0 (71.9, 80.1)	0.211
Height, m	1.80 (1.76, 1.85)	1.84 (1.80, 1.88)	1.81 (1.76, 1.86)	0.463
BMI, kg/m^2^	23.1 (22.1, 24.3)	23.6 (22.7, 24.4)	23.3 (22.4, 24.1)	0.775
Fat, %	19.0 (16.2, 21.7)	18.0 (15.4, 20.6)	19.9 (18.0, 21.9)	0.506
Appendicular lean mass, kg	27.6 (25.8, 29.3)	30.0 (27.5, 32.5)	27.7 (25.7, 29.6)	0.141
Lean body mass, kg	58.2 (55.0, 61.4)	63.1 (58.9, 67.3)	59.2 (55.5, 63.0)	0.116
Leg press 1RM, kg	308 (282, 334)	297 (262, 331)	297 (254, 339)	0.843
Leg extension 1RM, kg	114 (100, 129)	127 (109, 144)	123 (104, 143)	0.536
Chest press 1RM, kg	103 (91, 114)	104 (93, 116)	104 (91, 117)	0.969
Horizontal row 1RM, kg	80 (75, 86)	87 (77, 96)	79 (72, 86)	0.274
Vertical pull-down 1RM, kg	80 (72, 88)	85 (75, 96)	78 (71, 84)	0.365
Shoulder press 1RM, kg	111 (97, 125)	104 (91, 118)	104 (88, 121)	0.734

1 Values represent means and 95% CIs, *n* = 15 per group. Data were analyzed using a 1-factor ANOVA. 1RM, 1 repetition maximum.

### Pretesting

All subjects participated in a screening session, which was performed ≥1 wk prior to the experiment. First, subjects’ body mass and height were measured as well as body composition by DXA (Discovery A; Hologic). The system's software package (Hologic-Apex software version 4.5.3 with viewer software Hologic Physician's viewer, version 7.1) was used to determine whole body and regional lean and fat mass. In addition, participants were familiarized with the exercise equipment and performed maximum strength tests as determined by their 1 repetition maximum (1RM) for leg press, leg extension, chest press, horizontal row, vertical pull-down, and shoulder press, as described previously (11) (Table 1). The subjects were deemed healthy on the basis of their responses to a medical questionnaire.

### Diet and activity prior to the experiment

All subjects received a standardized dinner of the same composition (1900 kJ, providing 24.6 g protein, 69.3 g carbohydrate, and 19.1 g fat) the evening prior to the test day at the internal facility (Maastricht University Medical Centre+). Thereafter, all participants stayed overnight and slept at the internal facility. All volunteers refrained from alcohol and any sort of exhaustive physical labor and/or exercise 3 d prior to the experimental day. In addition, subjects were asked to report their dietary intake for 2 d prior to the experimental day. Energy and macronutrient intakes were calculated with the use of the Dutch Nutrients Database (NEVO-online version 2019/6.0; https://nevo-online.rivm.nl/).

### Study design

In the present study we selected “tracer naïve” subjects in a parallel group design, to prevent any potential impact of isotope tracer recycling. Each subject was randomly assigned to 1 of 3 experiments, in which the effect of postexercise ingestion of a low-protein control breakfast compared with raw eggs compared with boiled eggs on myofibrillar protein synthesis was studied. At the start of the experiment primed continuous l-[*ring*-^13^C_6_]-phenylalanine infusion was applied together with repeated blood sampling during the experimental day. After 2.5 h of rest, participants performed 15 min of cycling followed by 45 min of whole-body resistance-type exercise training. Thereafter a biopsy from musculus vastus lateralis was taken, before ingesting a low-protein control breakfast, 5 raw eggs, or 5 boiled eggs. In the subsequent 5-h recovery period, biopsies were taken from the vastus lateralis at *t* = 2 h and *t* = 5 h. Randomization was performed by using a computerized random-number generator.

### Experimental protocol

**FIGURE 1:**
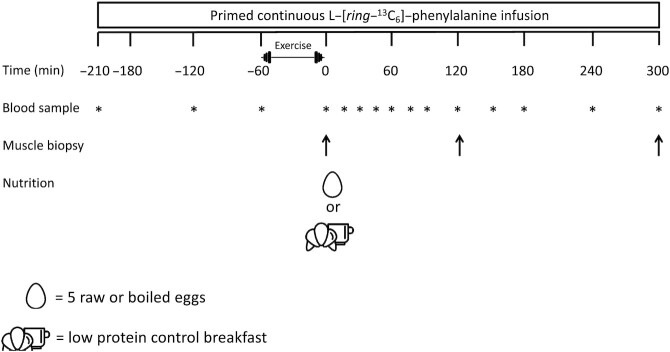
Schematic overview of the experimental protocol. Healthy young men performed whole-body resistance exercise and consumed either a low-protein control breakfast, 5 raw eggs, or 5 boiled eggs.

The experimental protocol is outlined in **Figure 1**. Each subject participated in 1 experiment. At the start of the experimental day at 08:00, following an overnight fast at our facilities, a polytetrafluoroethylene catheter was inserted into an antecubital vein for intravenous stable isotope tracer infusion. A second catheter was inserted in a dorsal hand vein of the contralateral arm, which was subsequently placed in a hot-box (60°C) for arterialized blood sampling. After baseline blood sample collection (*t* = −210 min), the plasma phenylalanine pool was primed with a single intravenous dose of l-[*ring*-^13^C_6_]-phenylalanine (2.25 μmol/kg). Subsequently, an intravenous infusion of l-[*ring*-^13^C_6_]-phenylalanine (infusion rate 0.05 μmol/kg/min) was initiated and maintained until the end of the trial using a calibrated IVAC 598 pump. During 2.5 h of supine rest, 2 additional arterialized blood samples (*t* = −120 and −60 min) were obtained. Subsequently, participants performed a whole-body resistance-type exercise session. After a 15-min warm-up on a cycle ergometer at self-selected intensity (88 W; 95% CI: 84, 91 W), subjects performed 4 sets of ∼8–10 repetitions (at 80% 1RM) on both the leg press and leg extension exercise followed by 2 sets of ∼8–10 repetitions (at 80% 1RM) on the chest press, horizontal row, vertical pull-down, and shoulder press. After completion of the exercise bout (*t* = 0 min), another arterialized blood sample was obtained together with a biopsy from the vastus lateralis muscle. Immediately afterwards, subjects ingested a control breakfast (protein content ∼5 g), 5 raw eggs, or 5 boiled eggs (both protein content of ∼30 g) at *t* = 0 min. Thereafter, repeated blood samples (*t* = 15, 30, 45, 60, 75, 90, 120, 150, 180, 240, and 300 min) were obtained together with biopsies from the vastus lateralis at *t* = 120 and *t* = 300 min. The biopsies were collected from the middle region of the vastus lateralis muscle (∼15 cm above the patella) with a Bergström needle under local anesthesia (12). The first 2 biopsies (at *t* = 0 and *t* = 120 min) were taken from the same leg. The last biopsy (*t* = 300 min) was collected from the contralateral leg. All biopsy samples were freed from any visible adipose tissue and blood, immediately frozen in liquid nitrogen, and stored at –80°C until subsequent analysis.

### Nutrition

Subjects ingested a low-protein control breakfast, 5 raw, or 5 boiled eggs (Albert Heijn) during the experimental infusion trial. The control breakfast consisted of a croissant (AH roomboter croissants; Albert Heijn), 10 g butter (Albert Heijn), and 350 mL orange juice (Jumbo). The control breakfast provided 1650 kJ, with 47 g carbohydrate, 20 g fat, and 5 g protein. Eggs were carefully weighed to ensure that every participant received a similar amount of boiled or raw eggs. The (hard) boiled eggs were steamed for ∼13 min with 65 mL water in an egg steamer. A total of 5 (un)boiled eggs provided 1400 kJ, with 0 g carbohydrate, 23 g fat, and 30 g protein. Participants were allowed to drink 300 mL of water with their eggs.

### Preparation of tracer

The stable isotope tracer l-[*ring-*^13^C_6_]-phenylalanine was purchased from Cambridge Isotopes and dissolved in 0.9% saline before infusion (Apotheek A15).

### Plasma and muscle tissue analysis

Blood samples (10 mL) were collected in EDTA-containing tubes and centrifuged at 1000× *g* and 4°C for 10 min. Aliquots of plasma were frozen in liquid nitrogen and stored at −80°C until analysis. Plasma glucose and insulin concentrations were analyzed using commercially available kits (GLUC3, Roche, ref: 05,168,791 190; and Immunologic, Roche, ref: 12,017,547 122, respectively). AA concentrations were determined by using ultraperformance LC-MS, as described previously (13). Plasma AA enrichments were determined by GC-MS (Agilent 7890A GC/5975C; MSD, Agilent Technologies). Myofibrillar protein-bound l-[*ring*-^13^C_6_]-phenylalanine enrichments were determined by GC-combustion-isotope-ratio-MS analysis (Thermo Fisher Scientific Delta V) as described in our previous work (13). For western blot analysis, a portion of each muscle sample frozen for biochemical analyses was taken and analyzed as described previously (14). In the present study, primary antibody dilution was 1:1000 and secondary antibody dilution was 1:10,000. All antibodies were purchased from Cell Signaling Technology; this included anti-phospho-mammalian target of rapamycin (mTOR) (Ser2448) (catalog number: 2971S), anti-mTOR (catalog number: 2972S), anti-phospho-S6K1 (Thr389) (catalog number: 9205 L), anti-phospho-S6K1 (Thr421/Ser424) (catalog number: 9204 L), anti-S6K1 (catalog number: 2217 L), anti-phosphoribosomal protein S6 (rpS6) (Ser240/Ser244) (catalog number: 2215 L), anti-phospho-rpS6 (Ser235/Ser236) (catalog number: 4856S), anti-rpS6 (catalog number: 2217 L), anti-phospho-eukaryotic translation initiation factor 4E-binding protein 1 (4E-BP1) (Thr37/46) (catalog number: 9459 L), and anti-4E-BP1 (catalog number: 9452 L).

### Calculations

Myofibrillar protein fractional synthetic rates (FSRs) were calculated by using the standard precursor-product equation, as follows:

(1) $$\begin{eqnarray*}
{\rm FSR} = \frac{{{\rm \Delta E }}_{\rm p}}{{\rm E}_{\rm precursor} \cdot {t}} \cdot {\rm 100}
\end{eqnarray*}$$

where ΔEp is the increment in myofibrillar protein-bound l-[*ring*-^13^C_6_]-phenylalanine enrichment after an incorporation period, E_precursor_ is the weighted mean plasma l-[*ring*-^13^C_6_]-phenylalanine enrichment during that incorporation period, and *t* is the incorporation period (hours). Weighted mean plasma enrichments were calculated by taking the average enrichment between all consecutive time points and correcting for the time between these sampling time points. The weighted mean plasma precursor pool is preferred in this setting, because the more frequent sampling time points allow for a more accurate correction of the transient changes in precursor pool enrichments over time (15). For basal (postabsorptive) FSRs, plasma protein samples at *t* = −210 min and muscle biopsy samples at *t* = 0 min were used; and for postprandial FSRs, muscle biopsy samples at *t* = 0, 120, and 300 min were used.

### Statistical analysis

A power calculation was performed with differences in postprandial myofibrillar protein FSR as the primary outcome measure with the use of an SD of 0.0065%/h in all treatments, and a difference in FSR of 0.008%/h between treatments (or ∼20% when expressed as relative difference between treatments). With a power of 80% and a significance level of 0.05, the final number of participants to be included was calculated as *n* = 12 per group. In order to account for potential dropouts and to ensure adequate power and ample data sets, we recruited 15 participants per group. All results are expressed as mean ± 95% CI. Baseline characteristics, dietary intake, and incremental area under the curve (iAUC) for integrated postprandial plasma AA concentrations above and below baseline (*t* = 0 min) were compared between groups using a 1-factor ANOVA. For time-dependent variables, a 2-factor (group × time) repeated-measures ANOVA with group as a between-subjects factor and time as a within-subjects factor was used (i.e., all time points for plasma concentrations, plasma AA enrichment, anabolic signaling, and basal compared with postprandial muscle data). In case of significant interactions, separate analyses were performed within each treatment group, as well as between treatment groups for every time period separately. In the case of significant group or time effects (when appropriate), Bonferroni post hoc analyses were performed to locate the effects. Cohen effect size (*d*) of the differences between raw eggs, control breakfast, and boiled eggs was calculated for the primary outcome (i.e., FSR) data. Effect sizes of 0.2 are considered small, 0.5 are considered medium, and 0.8 are considered large. Statistical significance was set at *P* ≤ 0.05. All calculations were performed using SPSS (version 26.0, IBM Corp).

## Results

### Dietary intake

Habitual dietary intakes are presented in **Table 2**. Analysis of the 2-d dietary intake records (collected in the 2 days prior to the experimental protocol) showed no differences in habitual food intake between groups. Daily protein intake in these subjects averaged 1.6 g/kg body mass/d in all 3 groups.

**TABLE 2:** Average 2-d habitual dietary intake of study participants^1^

	Control breakfast	Raw eggs	Boiled eggs	*P*
Energy, MJ/d	10.8 (8.5, 13.1)	12.0 (10.0, 13.9)	11.1 (9.5, 12.7)	0.664
Energy, kcal/d	2589 (2041, 3138)	2860 (2388, 3333)	2655 (2276, 3034)	0.664
Energy, kcal/kg BM/d	34.8 (27.1, 42.5)	35.8 (30.5, 41.0)	35.5 (29.4, 41.7)	0.973
Carbohydrate, g/d	279 (236, 321)	346 (281, 411)	295 (242, 347)	0.160
Carbohydrate, En%/d	47 (41, 53)	50 (47, 53)	46 (43, 50)	0.383
Fat, g/d	101 (59, 143)	97 (80, 115)	102 (86, 119)	0.962
Fat, En%/d	33 (28, 38)	31 (28, 34)	35 (31, 38)	0.312
Protein, g/d	123 (94, 151)	128 (103, 152)	120 (99, 142)	0.908
Protein, En%/d	20 (17, 24)	19 (16, 22)	19 (17, 22)	0.747
Protein, g/kg BM/d	1.6 (1.3, 2.0)	1.6 (1.3, 1.9)	1.6 (1.3, 1.9)	0.960

1 Values represent means and 95% CIs, *n* = 15 per group. Participants’ habitual energy, carbohydrate, fat, and protein intakes were calculated from a 2-d dietary record. Data were analyzed using a 1-factor ANOVA. BM, body mass; En%, percentage of energy.

### Plasma glucose and insulin

**FIGURE 2:**
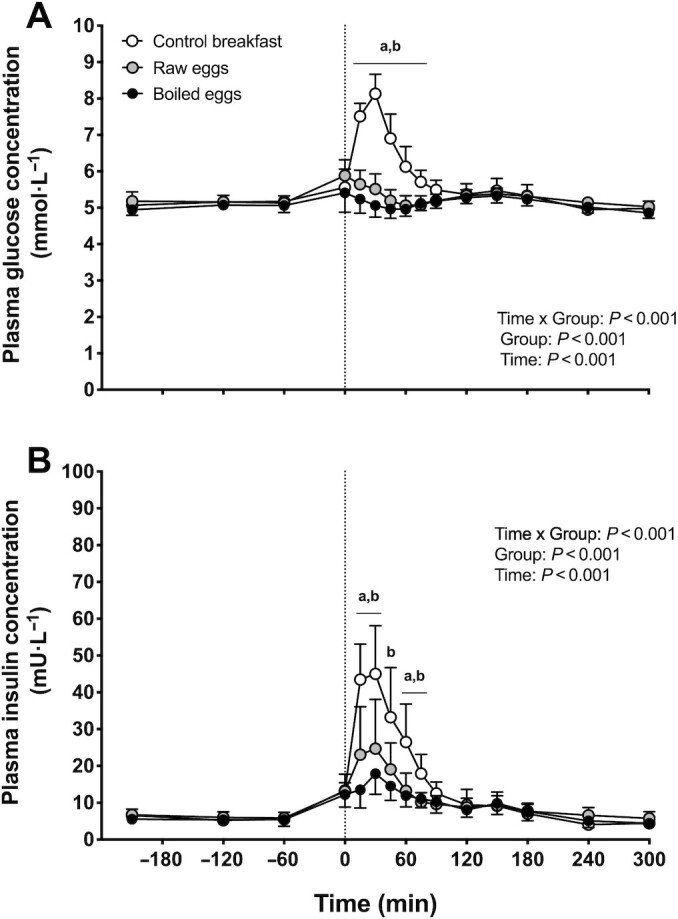
Plasma glucose (A) and insulin (B) concentrations over time after the ingestion of a low-protein control breakfast (*n* = 15), 5 raw eggs (*n* = 15), or 5 boiled eggs (*n* = 15) in healthy young males. Exercise was performed between *t* = −60 and 0 min. The dotted line represents the time of food ingestion. Values are mean + 95% CI. Data were analyzed with repeated measures (time × treatment group) ANOVA, and separate analyses were performed when a significant interaction was detected. Bonferroni post hoc testing was used to detect differences between groups: “a” denotes raw eggs were significantly different (*P* < 0.05) from control breakfast; “b” denotes boiled eggs were significantly different (*P* < 0.05) from control breakfast.

For both plasma glucose and insulin concentrations a main effect of time, a main effect of group, and a time × group interaction were observed (all *P* < 0.01). No significant differences were observed in fasting plasma glucose concentrations between groups (**Figure 2**A). Following food ingestion, a significant increase in plasma glucose concentrations was observed in the control breakfast group only (up to *t* = 45 min; *P* < 0.005), with higher plasma glucose concentrations (up to *t* = 75 min) in the control breakfast group compared with both the boiled eggs and the raw eggs groups (*P* < 0.005).

Plasma insulin concentrations (Figure 2B) were significantly increased immediately after exercise in all 3 groups (*P* < 0.005), with no significant differences being observed in plasma insulin concentrations between groups prior to food ingestion. Postprandial plasma insulin concentrations were significantly increased in the control breakfast group only (up to *t* = 60 min; *P* < 0.05), with higher plasma insulin concentrations in the control breakfast group compared with both the boiled eggs and the raw eggs groups (*P* < 0.05).

### Plasma AAs

**FIGURE 3:**
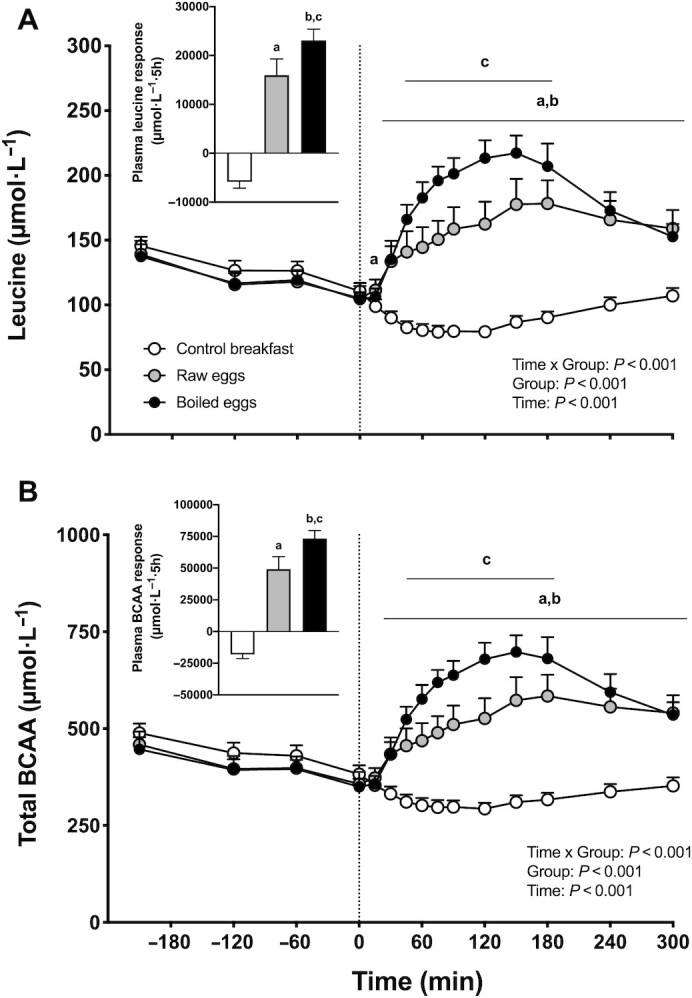
Plasma leucine (A), and total BCAA (B) concentrations over time, and total leucine (panel A insert) and BCAA (panel B insert) responses, expressed as iAUCs, following the ingestion of a low-protein control breakfast (*n* = 15), 5 raw eggs (*n* = 15), or 5 boiled eggs (*n* = 15) in healthy young males. Exercise was performed between *t* = −60 and 0 min. The dotted line represents the time of food ingestion. Values are mean + 95% CI. For plasma concentrations over time, data were analyzed with repeated measures (time × treatment group) ANOVA, and separate analyses were performed when a significant interaction was detected. The iAUC data were analyzed with a 1-factor ANOVA. Bonferroni post hoc testing was used to detect differences between groups. For iAUC data, a group effect was observed, *P* < 0.001. “a” denotes raw eggs were significantly different (*P* < 0.05) from control breakfast; “b” denotes boiled eggs were significantly different (*P* < 0.05) from control breakfast; “c” denotes boiled eggs were significantly different (*P* < 0.05) from raw eggs. BCAA, branched-chain amino acid; iAUC, incremental area under the curve.

Plasma leucine and total branched-chain amino acid (BCAA) concentrations are depicted in **Figure 3**. Significant time × group interactions were observed for both plasma leucine and total BCAA concentrations (*P <* 0.001). In addition, a significant group effect was observed for the iAUC for both leucine and total BCAA concentrations (*P <* 0.001). Postprandial plasma leucine (Figure 3A) and BCAA (Figure 3B) concentrations significantly increased from baseline values (*t* = 0 min) onwards and remained elevated throughout the entire postprandial period in both the raw eggs and boiled eggs groups (*P <* 0.005). As a result, postprandial plasma leucine and BCAA concentrations were significantly higher in both the raw eggs and boiled eggs groups compared with the control breakfast group (*P <* 0.05). Postprandial plasma leucine and BCAA concentrations showed a greater increase following boiled eggs compared with raw eggs ingestion, with significantly higher values between *t* = 45 and 180 min in the boiled eggs group compared with the raw eggs group (*P <* 0.05). In agreement, the postprandial plasma leucine (Figure 3A insert) and BCAA (Figure 3B insert) iAUC were significantly greater in the raw eggs and boiled eggs groups compared with the control breakfast group (*P <* 0.001), with higher values following ingestion of boiled compared with raw eggs (*P <* 0.001).

**FIGURE 4:**
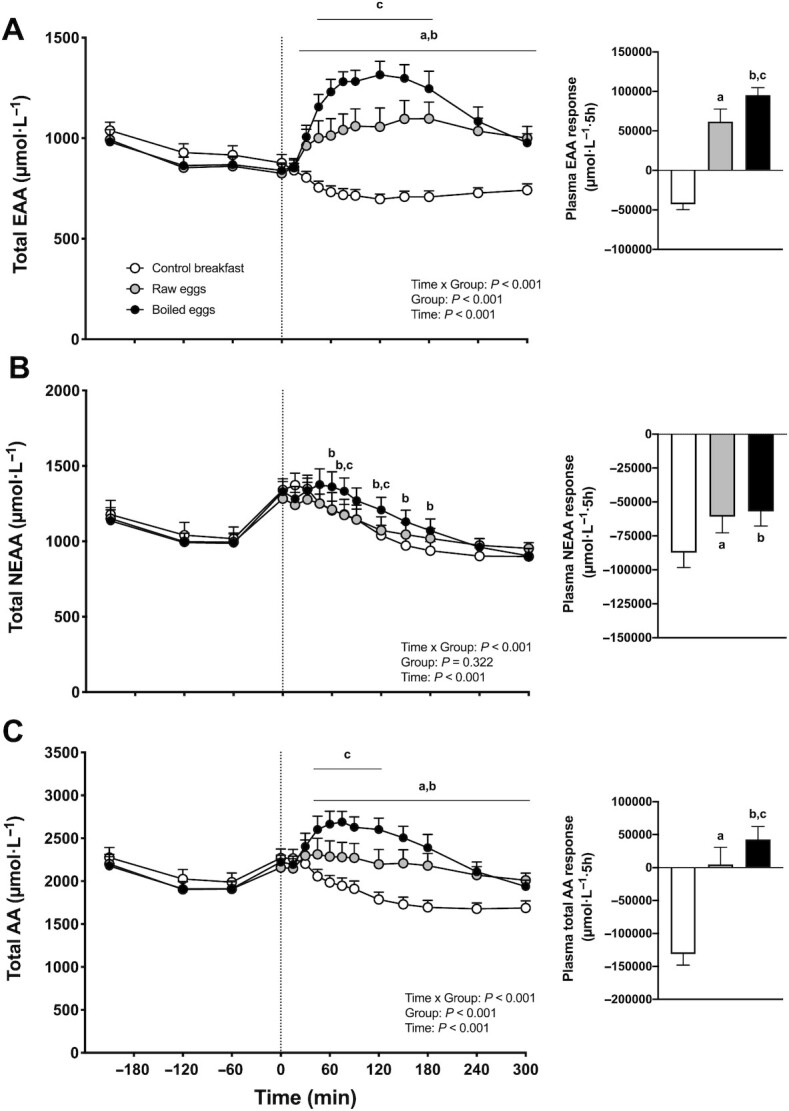
Plasma total EAA (A), NEAA (B), and AA (C) concentrations over time, and total EAA (panel A insert), NEAA (panel B insert), and AA (panel C insert) responses, expressed as iAUCs, after the ingestion of a low-protein control breakfast (*n* = 15), 5 raw eggs (*n* = 15), or 5 boiled eggs (*n* = 15) in healthy young males. Exercise was performed between *t* = −60 and 0 min. The dotted line represents the time of food ingestion. Values are mean + 95% CI. For plasma concentrations over time, data were analyzed with repeated measures (time × treatment group) ANOVA and separate analyses were performed when a significant interaction was detected. The iAUC data were analyzed with a 1-factor ANOVA. Bonferroni post hoc testing was used to detect differences between groups. For iAUC data, a group effect was observed, *P* < 0.001. “a” denotes raw eggs were significantly different (*P* < 0.05) from control breakfast; “b” denotes boiled eggs were significantly different (*P* < 0.05) from control breakfast; “c” denotes boiled eggs were significantly different (*P* < 0.05) from raw eggs. AA, amino acid; EAA, essential amino acid; iAUC, incremental area under the curve; NEAA, nonessential amino acid.

Plasma total essential amino acid (EAA), total nonessential amino acid (NEAA-glutamine), and total AA (AA-glutamine) concentrations are depicted in **Figure 4**. Significant time × group interactions were observed for plasma total EAA, total NEAA, and total AA concentrations (*P <* 0.001). In addition, a significant group effect was observed for the iAUC for total EAA, total NEAA, and total AA concentrations (*P <* 0.001). Postprandial plasma EAA, NEAA, and total AA concentrations decreased in the control breakfast group, with significantly lower values compared with baseline values throughout the entire postprandial period (*P <* 0.05). In both the raw eggs and boiled eggs groups, postprandial plasma EAA concentrations were significantly higher and postprandial plasma NEAA concentrations were significantly lower compared with baseline values (*P <* 0.05). For total plasma AA concentrations, a significant increase was observed only in the boiled eggs group (*P* < 0.001). Postprandial plasma EAA and total AA concentrations were significantly higher in both the raw eggs and boiled eggs groups compared with the control breakfast group (*P <* 0.05), with the boiled eggs group showing significantly higher postprandial plasma EAA and total AA concentrations compared with the raw eggs group (*P <* 0.05). In agreement, the iAUCs of postprandial plasma EAA (Figure 4A insert) and total AA (Figure 4C insert) concentrations were significantly greater in the raw eggs and boiled eggs groups compared with the control breakfast group (*P <* 0.001) and higher in the boiled eggs group compared with the raw eggs group (*P <* 0.05). Postprandial plasma NEAA concentrations were significantly higher in the boiled eggs group compared with the control breakfast group (*P <* 0.001), with no differences between the control breakfast and the raw eggs groups. Postprandial plasma NEAA concentrations were significantly higher in the boiled eggs group compared with the raw eggs group at *t* = 75 and 120 min (*P <* 0.05). The iAUCs of postprandial plasma NEAA concentrations (Figure 4B insert) were significantly different in the raw eggs and boiled eggs groups compared with the control breakfast group (both *P <* 0.005), with no differences observed between the boiled eggs and raw eggs groups (*P >* 0.05).

### Isotope tracer analysis

**FIGURE 5:**
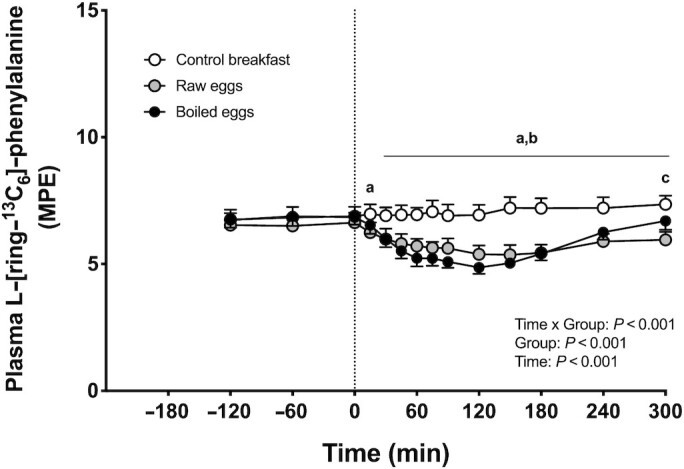
Plasma l-[*ring*-^13^C_6_]-phenylalanine enrichments over time after the ingestion of a low-protein control breakfast (*n* = 15), 5 raw eggs (*n* = 15), or 5 boiled eggs (*n* = 15) in healthy young males. Exercise was performed between *t* = −60 and 0 min. The dotted line represents the time of food ingestion. Values are mean + 95% CI. Data were analyzed with repeated measures (time × treatment group) ANOVA, and separate analyses were performed when a significant interaction was detected. Bonferroni post hoc testing was used to detect differences between groups. “a” denotes raw eggs were significantly different (*P* < 0.05) from control breakfast; “b” denotes boiled eggs were significantly different (*P* < 0.05) from control breakfast; “c” denotes boiled eggs were significantly different (*P* < 0.05) from raw eggs. MPE, mole % excess.

A significant time × group interaction was observed for plasma l-[*ring*-^13^C_6_]-phenylalanine enrichments (*P <* 0.001). Prior to food ingestion, plasma l-[*ring*-^13^C_6_]-phenylalanine enrichments averaged 6.8 (95% CI: 6.5, 7.1), 6.5 (95% CI: 6.2, 6.9), and 6.8 (95% CI: 6.5, 7.1) mole % excess (MPE) in the control breakfast, raw eggs, and boiled eggs groups, respectively, with no differences over time or between groups (**Figure 5**). Postprandial plasma l-[*ring*-^13^C_6_]-phenylalanine enrichments (compared with baseline, *t* = 0 min)) were significantly lower in the raw and boiled eggs groups during the postprandial period (*P <* 0.05). Plasma l-[*ring*-^13^C_6_]-phenylalanine enrichments during the entire postprandial period averaged 7.1 (95% CI: 6.8, 7.4), 5.7 (95% CI: 5.5, 6.0), and 5.7 (95% CI: 5.4, 6.0) MPE for the control breakfast, raw eggs, and boiled eggs groups, respectively, with significantly lower values in the raw and boiled eggs groups compared with the control breakfast group (*P* < 0.05).

**FIGURE 6:**
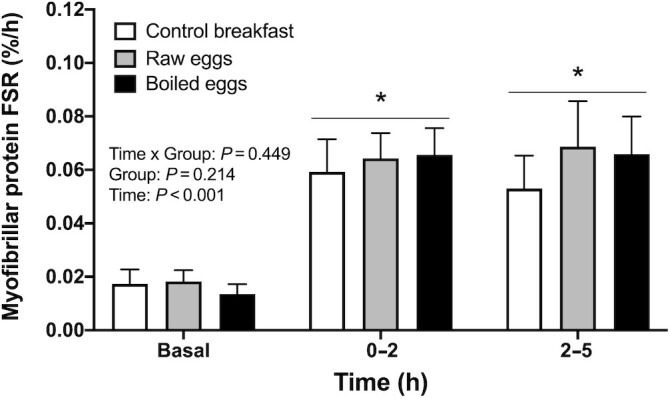
Myofibrillar protein fractional synthetic rates (FSR; in %/h) during the fasted state (basal) and over the early (0–2 h), and late (2–5 h) postexercise and postprandial period after the ingestion of a low-protein control breakfast (*n* = 15), 5 raw eggs (*n* = 15), or 5 boiled eggs (*n* = 15) in healthy young males. Values are mean + 95% CI. Data were analyzed with repeated measures (time × treatment group) ANOVA. *Denotes significant time effect (*P* < 0.001).

**FIGURE 7:**
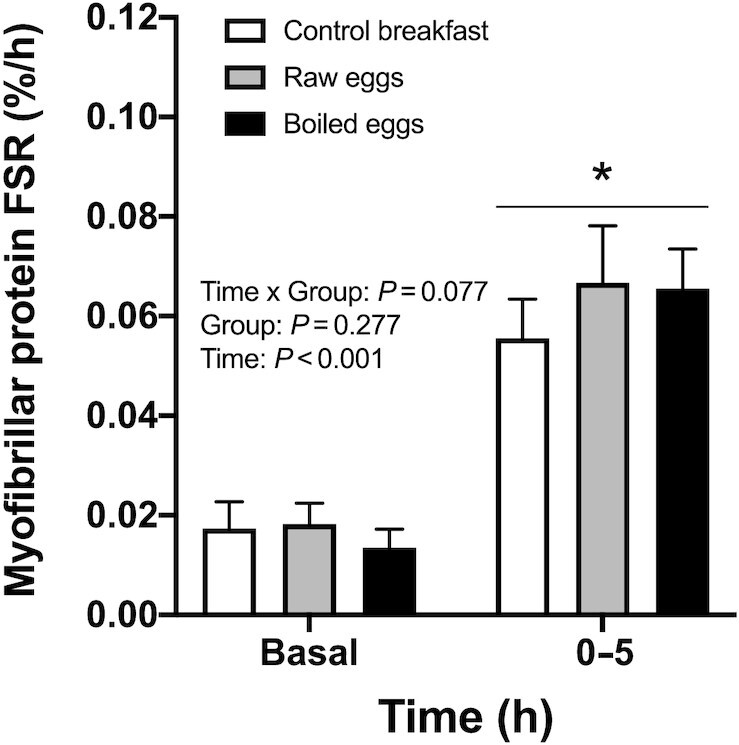
Myofibrillar protein fractional synthetic rates (FSR; in %/h) during the fasted state (basal) and over the entire (0–5 h) postexercise and postprandial period after the ingestion of a low-protein control breakfast (*n* = 15), 5 raw eggs (*n* = 15), or 5 boiled eggs (*n* = 15) in healthy young males. Values are mean + 95% CI. Data were analyzed with repeated measures (time × treatment group) ANOVA. *Denotes significant time effect (*P* < 0.001); there was a trend for a significant interaction effect (*P* = 0.077).

Myofibrillar protein synthesis rates calculated based on the plasma precursor pool are depicted in **Figures 6** and **7**. Myofibrillar protein synthesis rates increased following food ingestion in all groups. From basal fasting values to the early 0–2-h postprandial period, myofibrillar protein synthesis rates increased from 0.017%/h (95% CI: 0.012, 0.023%/h) to 0.059%/h (95% CI: 0.047, 0.071%/h), from 0.018%/h (95% CI: 0.014, 0.022%/h) to 0.064%/h (95% CI: 0.055, 0.074%/h), and from 0.013%/h (95% CI: 0.010, 0.017%/h) to 0.066%/h (95% CI: 0.056, 0.076%/h), in the control breakfast, raw eggs, and boiled eggs groups, respectively (*P* < 0.001; Figure 6). Myofibrillar protein synthesis rates remained elevated during the late (2–5-h) postprandial period in all groups, with values of 0.053%/h (95% CI: 0.041, 0.065%/h), 0.069%/h (95% CI: 0.052, 0.086%/h), and 0.066%/h (95% CI: 0.052, 0.080%/h) in the control breakfast, raw eggs, and boiled eggs groups, respectively (*P* < 0.001). Throughout the entire 0–5-h postprandial period, myofibrillar protein synthesis rates were elevated above basal values (main effect of time: *P* < 0.001; Figure 7) and were ∼20% higher following ingestion of the raw eggs (0.067%/h; 95% CI: 0.056, 0.077%/h) and boiled eggs (0.065%/h; 95% CI: 0.058, 0.073%/h) compared with the control breakfast group (0.056%/h; 95% CI: 0.048, 0.063%/h). Although this difference did not reach statistical significance (time × group interaction: *P* = 0.077), meaningful effect sizes were observed between the egg groups and the control breakfast group (*d* for raw eggs compared with control breakfast = 0.63; *d* for boiled eggs compared with control breakfast = 0.69), but not between both egg groups (*d* = 0.07).

### Anabolic signaling

The phosphorylation status (ratio of phosphorylated to total protein) of key proteins involved in the initiation of muscle protein synthesis is presented in **Supplemental Figure 2**A–F. No significant time effect, group effect, or time × group interactions were observed for muscle mTOR (Ser2448) (Supplemental Figure 2A) and p70S6K (Thr389) (Supplemental Figure 2B) phosphorylation status. A significant time effect was observed for muscle p70S6K (Thr421/Ser424) (Supplemental Figure 2C), rpS6 (Ser235/236) (Supplemental Figure 2E), and 4E-BP1 (Thr37/46) (Supplemental Figure 2F) phosphorylation status (*P* < 0.05), with no significant group effects or time × group interactions observed. For muscle p70S6K (Thr421/Ser424), time points *t* = 2 and 5 h were significantly lower compared with *t* = 0 h (*P* < 0.001; Supplemental Figure 2C). For muscle rpS6 (Ser235/236), time point *t* = 2 h was significantly lower compared with *t* = 0 h (*P* = 0.033; Supplemental Figure 2E); and for muscle 4E-BP1 (Thr37/46) *t* = 5 h was significantly higher compared with *t* = 2 h (*P* = 0.036; Supplemental Figure 2F). A significant group effect was observed for muscle rpS6 (Ser240/244) phosphorylation status (*P* = 0.012; Supplemental Figure 2D) with boiled eggs higher compared with raw eggs (*P* = 0.010), with no significant time effect or time × group interaction observed.

## Discussion

In the present study we observed that ingestion of 30 g protein provided as 5 eggs significantly increased circulating plasma EAA concentrations, with a greater rise in circulating AAs following the ingestion of 5 boiled compared with 5 unboiled, raw eggs. Despite the more pronounced rise in postprandial plasma AA responses after the ingestion of boiled compared with raw eggs, we observed no significant differences in postprandial myofibrillar protein synthesis rates throughout 5 h of postexercise recovery.

Eggs represent a high-quality protein source based upon a high and well-balanced EAA content (well above the WHO/FAO/United Nations University requirements for adults) and a highly digestible indispensable amino acid score (16, 17). Not surprisingly, ingestion of egg protein has been shown to robustly increase postexercise muscle protein synthesis rates (18, 19) with maximal muscle protein synthesis rates observed after ingesting 20–40 g protein (19). When consumed as whole foods, eggs are generally cooked or boiled prior to consumption. This heat treatment can significantly impact postprandial protein digestion and subsequent AA absorption kinetics (2, 3). Here, we compared the postprandial rise in circulating AAs following ingestion of 5 boiled compared with 5 unboiled, raw eggs in healthy young males recovering from a bout of exercise (Figures 3 and 4). Following egg ingestion, we demonstrated an increase in plasma leucine, BCAA, and EAA concentrations, with greater increases after ingesting boiled when compared with raw eggs. These results are in line with previous pioneering work by Evenepoel et al. (2, 3), who showed that cooked eggs have a much higher digestibility (∼91%) when compared with raw eggs (∼51%). The greater digestibility of cooked eggs has been attributed to the denaturation of the protein, resulting in structural changes of the different egg proteins (e.g., ovalbumin, ovotransferrin) that facilitate the hydrolytic action of digestive enzymes (20). Furthermore, heating can also (partly) inactivate the effect of different trypsin inhibitors present within the egg (21). Consequently, given its attenuated protein digestion, AA absorption, and subsequent postprandial rise in circulating AAs, it could be speculated that ingesting raw (compared with boiled) eggs would compromise the postprandial stimulation of muscle protein synthesis.

In the present study we observed that myofibrillar protein synthesis rates were significantly increased during the early (0–2 h), late (2–5 h), and total (0–5 h) postexercise period when compared with basal, resting values (Figures 6 and 7). These data confirm numerous observations showing increases in muscle protein synthesis rates after a bout of exercise (22–27). No significant differences were observed between groups during 5 h of postexercise recovery (*P* = 0.077); however, we did observe ∼20% higher postprandial myofibrillar protein synthesis rates following the ingestion of boiled or raw eggs when compared with the control breakfast (Figure 7). In addition, meaningful effect sizes of 0.6–0.7 were observed between the egg groups and the control breakfast group. Taken together, our results support the contention that adequate (≥20 g) high-quality protein intake further increases postexercise muscle protein synthesis rates (19, 28–31). However, in contrast to our hypothesis, differences in postprandial AA responses after the ingestion of 5 boiled compared with unboiled, raw eggs did not translate into differences in postexercise muscle protein synthesis rates.

We can only speculate on the factors responsible for the absence of differences in postexercise muscle protein synthesis rates after the ingestion of boiled compared with raw eggs, in spite of the obvious differences in postprandial plasma AA availability. However, it seems evident that the provision of 5 eggs already provided more protein (30 g) than the amount previously reported to be required to maximize postexercise myofibrillar protein synthesis rates (19, 31, 32). In support, our results are consistent with recent work showing that higher plasma AA availability does not further stimulate muscle protein synthesis when ample amounts of protein are ingested during recovery from resistance-type exercise (33). Furthermore, several other studies have failed to show differences in postexercise muscle protein synthesis when ingesting ample amounts of different protein sources (despite substantial differences in the magnitude of postprandial aminoacidemia induced) (34–41). We can only speculate whether differential responses could be expected when fewer eggs would have been ingested. This might not be of relevance in a postexercise setting where ample protein is typically ingested. However, in situations where smaller amounts of protein are ingested by more clinically compromised individuals, the level of processing could become more relevant as a factor responsible for determining the anabolic properties of a meal.

Because resistance-type exercise and dietary protein intake activate intramuscular signaling proteins that regulate protein translation-initiation, we also assessed several targets of the mTOR pathway (i.e., mTOR, p70S6 kinase, rpS6, and 4E-BP1) to assess potential differences between treatment groups. In the current study we did not observe any significant differences between groups over time (Supplemental Figure 2). This is not necessarily surprising because we only collected muscle tissue biopsies during recovery from exercise and, as such, most of the signaling responses were likely already strongly activated in the time period leading up to the collection of the first biopsy (18). The fact that no differences were observed in anabolic signaling between the different groups during postexercise recovery is in line with recent findings showing that differences in aminoacidemia do not necessarily appear to influence anabolic signaling when assessed during recovery from exhaustive exercise (33). Overall, the current findings in anabolic signaling agree with the absence of differences in postprandial muscle protein synthesis rates between groups.

Because postexercise muscle protein synthesis rates did not differ following the ingestion of 5 boiled compared with raw eggs, it is evident that Rocky did not compromise his skeletal muscle adaptive response to exercise by not boiling his eggs. However, it should be noted that there could still be other reasons why people want to consider boiling eggs prior to consumption. One obvious reason is for general taste and texture preferences. Another good reason could be to eliminate the risk of salmonella infection. Even though it appears that only a small proportion (<0.06%) of eggs are contaminated with salmonella and thus pose a low infection risk when eating raw eggs (42), this risk will be fully eliminated when the eggs are properly boiled prior to consumption (43).

In conclusion, ingestion of 5 raw, unboiled eggs as opposed to 5 boiled eggs attenuates the postprandial rise in circulating EAA concentrations, but does not compromise the postprandial increase in myofibrillar protein synthesis rates during recovery from exercise. Though Rocky was not aware of the benefits of boiling eggs to postprandial protein handling, he did not compromise postexercise muscle conditioning by consuming his eggs unboiled.

## Supplementary Material

nxac174_Supplemental_Files

## Data Availability

Data described in the manuscript (in deidentified form), code book, and analytic code will be made available upon request pending application and approval from the corresponding author.

## Notes

This study was internally funded by Maastricht University.

Author disclosures: LJCvL has received research grants, consulting fees, speaking honoraria, or a combination of these for research on the impact of exercise and nutrition on postprandial protein metabolism. A full overview on research funding is provided at https://www.maastrichtuniversity.nl/l.vanloon. LBV has received research funding from FrieslandCampina BV (not related to the work presented here). All other authors report no conflicts of interest.

Supplemental Figures 1 and 2 are available from the “Supplementary data” link in the online posting of the article and from the same link in the online table of contents at https://academic.oup.com/jn/.

CJF and WJHH contributed equally to this study.

Abbreviations used: AA, amino acid; BCAA, branched-chain amino acid; EAA, essential amino acid; FSR, fractional synthetic rate; iAUC, incremental area under the curve; MPE, mole % excess; mTOR, mammalian target of rapamycin; NEAA, nonessential amino acid; 1RM, 1 repetition maximum.
