# Supplementary material for: Raw Eggs To Support Postexercise Recovery in Healthy Young Men: Did Rocky Get It Right or Wrong?
Source: J Nutr. 2022 Aug 9;152(11):2376–86. doi: 10.1093/jn/nxac174 (PMC9644172; doi:10.1093/jn/nxac174)
Supplement: nxac174_Supplemental_Files [file nxac174_supplemental_files.zip › Supplementary_Figure_1.pptx]

## Slide 1
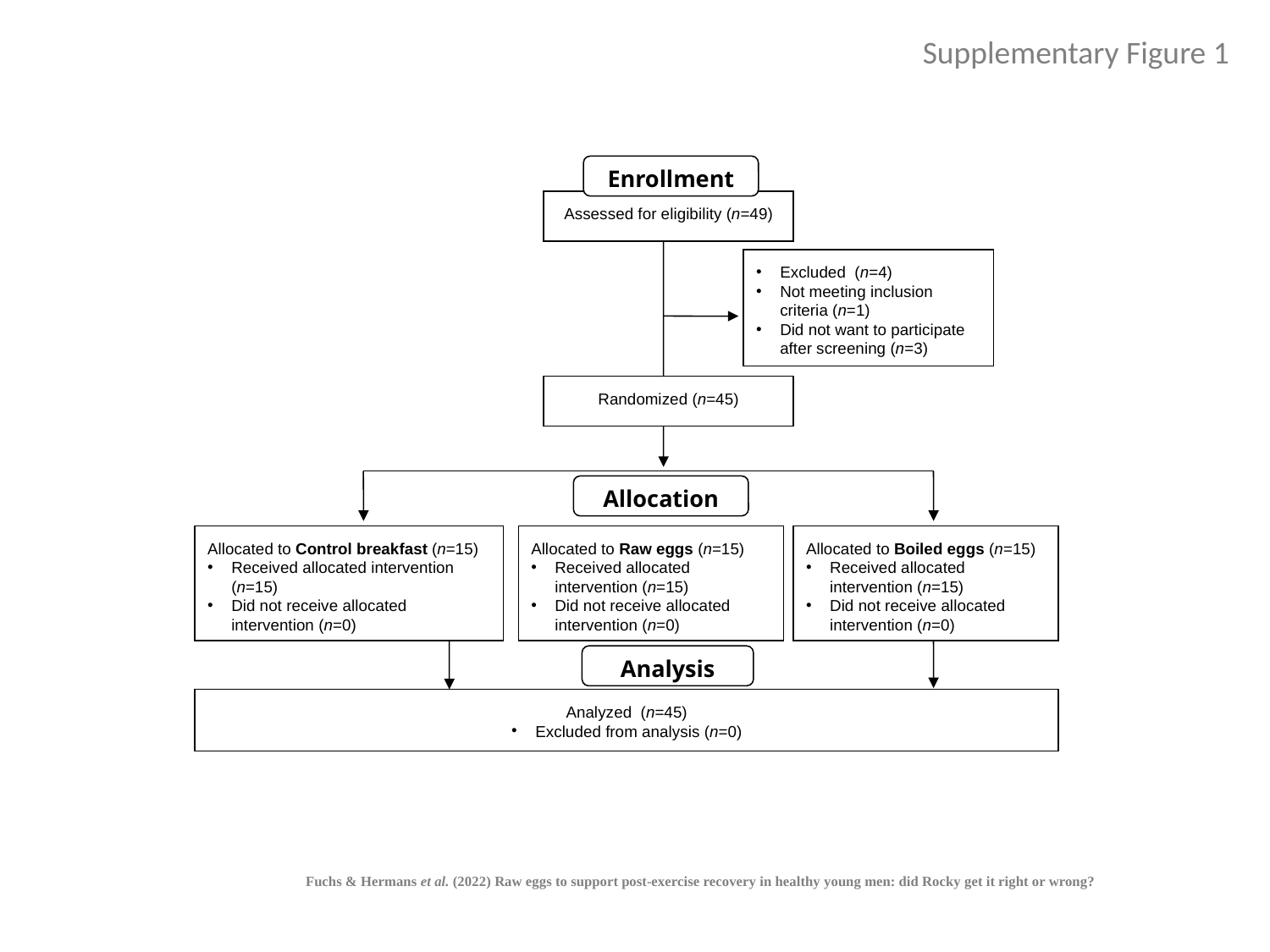

Supplementary Figure 1
Enrollment
Assessed for eligibility (n=49)
Excluded (n=4)
Not meeting inclusion criteria (n=1)
Did not want to participate after screening (n=3)
Randomized (n=45)
Allocation
Allocated to Raw eggs (n=15)
Received allocated intervention (n=15)
Did not receive allocated intervention (n=0)
Allocated to Boiled eggs (n=15)
Received allocated intervention (n=15)
Did not receive allocated intervention (n=0)
Analysis
Analyzed (n=45)
Excluded from analysis (n=0)
Allocated to Control breakfast (n=15)
Received allocated intervention (n=15)
Did not receive allocated intervention (n=0)
Fuchs & Hermans et al. (2022) Raw eggs to support post-exercise recovery in healthy young men: did Rocky get it right or wrong?
