# Supplementary material for: Raw Eggs To Support Postexercise Recovery in Healthy Young Men: Did Rocky Get It Right or Wrong?
Source: J Nutr. 2022 Aug 9;152(11):2376–86. doi: 10.1093/jn/nxac174 (PMC9644172; doi:10.1093/jn/nxac174)
Supplement: nxac174_Supplemental_Files [file nxac174_supplemental_files.zip › Supplementary_Figure_2_Legend_for_publication.docx]

**SUPPLEMENTARY FIGURE 2 LEGEND**

**Supplementary Figure 2**. Skeletal muscle phosphorylation status (ratio of phosphorylated to total protein) of mTOR (Ser2448) (**A**), p70S6K (Thr389) (**B**), p70S6K (Thr421/Ser424) (**C**), rpS6 (Ser240/244) (**D**), rpS6 (Ser235/236) (**E**), and 4E-BP1 (Thr37/46) (**F**) immediately after exercise (*t*= 0 h) and at *t*= 2 and 5 h after the ingestion of a low protein control breakfast (*n* = 15), 5 raw eggs (*n* = 15), or 5 boiled eggs (*n* = 15) in healthy young males. Values are mean + 95% CI. Data were analyzed with repeated measures (time X treatment group) ANOVA. No significant interaction was detected.

The blots in this figure were quantified on an Odyssey scanner using Image Studio Version 5.2.4 software. This software automatically detects saturated pixels, given in blue when using the grey-white scale. Quantification was performed in such a way that there were no pixels saturated. Therefore, overexposure was totally eliminated in this quantification and could not occur. No infinity signals were detected by the software. This was applied to the phospho blots as well as to the non-phospho blots.
