# Supplementary figures and images for: Raw Eggs To Support Postexercise Recovery in Healthy Young Men: Did Rocky Get It Right or Wrong?
Source: J Nutr. 2022 Aug 9;152(11):2376–86. doi: 10.1093/jn/nxac174 (PMC9644172; doi:10.1093/jn/nxac174)

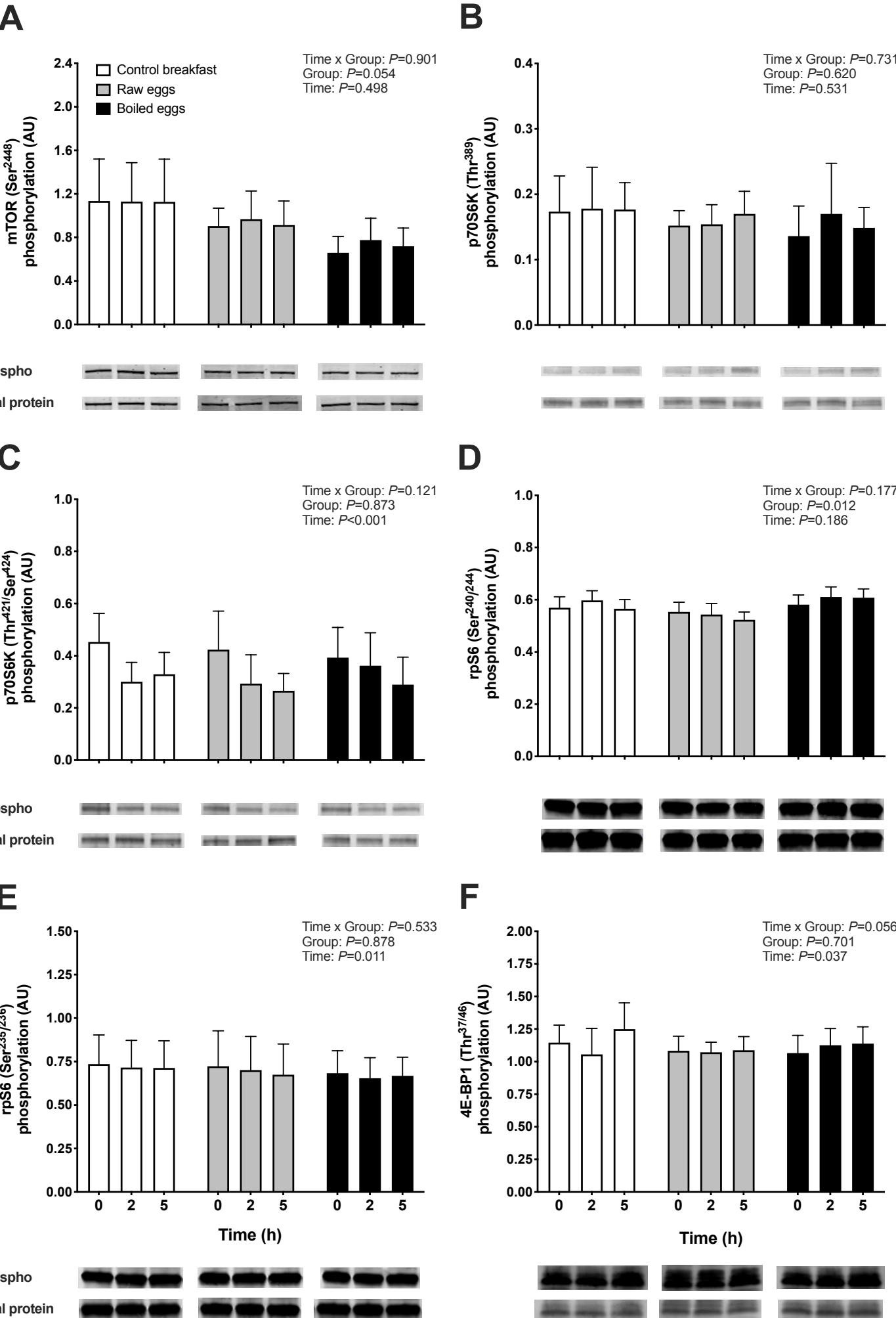

Supplement: nxac174_Supplemental_Files [file nxac174_supplemental_files.zip › Supplementary_Figure_2.pdf]
